# High Free IgE and Mast Cell Activation in Long COVID: Mechanisms of Persistent Immune Dysregulation

**DOI:** 10.3390/life15101538

**Published:** 2025-10-01

**Authors:** Sylvia Genova, Mina Pencheva, Hasan Burnusuzov, Martina Bozhkova, Georgi Kulinski, Stefka Kostyaneva, Eduard Tilkiyan, Tsvetana Abadjieva

**Affiliations:** 1 Department of General and Clinical Pathology, Faculty of Medicine, Medical University of Plovdiv, 4002 Plovdiv, Bulgaria; 2 Department of Medical Physics and Biophysics, Faculty of Pharmacy, Medical University of Plovdiv, 4002 Plovdiv, Bulgaria; 3 Department of Medical Microbiology and Immunology, Faculty of Medicine, Medical University of Plovdiv, 4002 Plovdiv, Bulgaria; hasan.burnusuzov@mu-plovdiv.bg (H.B.); martina.bozhkova@mu-plovdiv.bg (M.B.); 4 Research Institute, Medical University of Plovdiv, 4002 Plovdiv, Bulgaria; 5 Gastroenterology Section, Second Department of Internal Medicine, Faculty of Medicine, Medical University of Plovdiv, 4002 Plovdiv, Bulgaria; georgi.kulinski@mu-plovdiv.bg; 6 Nephrology Section, Second Department of Internal Medicine, Faculty of Medicine, Medical University of Plovdiv, 4002 Plovdiv, Bulgaria; stefka.kostyaneva@mu-plovdiv.bg (S.K.); eduard.tilkiyan@mu-plovdiv.bg (E.T.); 7 Department of Dermatology and Venereology, Faculty of Medicine, Medical University of Plovdiv, 4002 Plovdiv, Bulgaria; tsvetana.abadjieva@mu-plovdiv.bg

**Keywords:** COVID-19, IgE, mast cells, basophils

## Abstract

**Background:** Elevated serum IgE has been reported in severe COVID-19, suggesting that mast cell activation, allergic-like responses, and possible viral immune evasion occur. **Objective:** This study aimed to assess serum IgE, IgG, eosinophils, basophils, IL-10, and IL-33 in COVID-19 patients, and evaluate the infiltration of mast cells, basophils, and plasma cells in fatal cases. **Methods:** This retrospective study included 21 patients with severe COVID-19 or related respiratory conditions hospitalized in Plovdiv, Bulgaria (February 2020–May 2022). Serum immunoglobulins were quantified via immunoassays; IL-10 and IL-33 were also measured. Lung tissues from 30 autopsies were examined histologically and immunohistochemically using CD117 (mast cells) and CD138 (plasma cells). **Results:** Elevated IgE (>100 IU/mL) occurred in 10/21 patients, with two patients exhibiting levels exceeding 1000 IU/mL. High IgE correlated with reduced eosinophils and basophils, except in post-COVID lobar pneumonia. IL-10 was significantly increased, while IL-33 was reduced in acute and long COVID. Lung histology showed the accumulation of mast cells and plasma cells (5–20/field) during the diffuse alveolar damage and acute respiratory distress syndrome (ARDS) phases, but not in later fibrotic stages. Basophils are located near capillary basement membranes and the endothelium. **Conclusions:** SARS-CoV-2 may induce IgE-driven allergic-like mechanisms that contribute to severity. Monitoring IgE and mast cell activity may provide prognostic and therapeutic value, while elevated IgG4 could mitigate the effects of IgE.

## 1. Introduction

Three years after the official declaration of the end of the COVID-19 pandemic, research continues to investigate the mechanisms underlying severe pulmonary injury in patients with SARS-CoV-2 infection. Recent analyses aimed at identifying risk factors for disease progression have found that elevated total serum immunoglobulin E (IgE) and C-reactive protein (CRP) levels are independent predictors of worse outcomes. Patients with clinically deteriorating COVID-19 present with significantly higher total IgE concentrations and markedly lower serum eosinophil counts compared with stable patients [1].

Emerging evidence indicates that IgE-mediated pathways may contribute not only to acute lung injury but also to prolonged post-COVID immune dysregulation, potentially explaining the persistent respiratory symptoms observed in some patients [2]. Cytokines are thought to drive B-cell proliferation, immunoglobulin class switching to IgE, and the degranulation of mast cells and basophils. Type 2 inflammation is characterized by tissue infiltration with eosinophils and basophils, followed by the degranulation of these cells [3]. Mast cells and basophils, when activated by IgE, release a variety of inflammatory mediators, including histamine, proteases, and cytokines, which can exacerbate alveolar damage and vascular leakage [4,5].

In our previous study, we demonstrated that large amounts of IgG4 accumulate in the lung parenchyma during the acute stage of COVID-19 [6]. Rubio-Casillas et al. (2025) expanded on this observation, suggesting that the increase in IgG4 may represent a compensatory response to the elevated production of IgE by basophils and mast cells [7,8]. Immunoglobulin G4 (IgG4) is an anti-inflammatory antibody with a limited capacity to trigger effective immune responses, and may mitigate allergic reactions by blocking IgE activity [7,8,9,10]. Furthermore, the dynamic interplay between IgE and IgG4 suggests a complex regulatory mechanism, where IgG4 may attempt to mitigate IgE-driven inflammation but could simultaneously impair effective viral clearance [11,12,13].

Recent studies have revealed an unexpected IgE response directed against the receptor-binding domain (RBD) and other SARS-CoV-2 proteins; this was observed after both natural infection and vaccination [14,15,16,17]. The concurrent presence of IgE and IgG4 in COVID-19 suggests that the virus may trigger a so-called allergic-like immune response as a means of evading immune surveillance [7,18]. Understanding these interactions could reveal new biomarkers for disease severity and highlight potential therapeutic targets aimed at modulating allergic-like immune responses in COVID-19. Finally, the observation of tissue-specific IgE deposition in the lungs underscores the importance of considering local immune phenomena in addition to systemic measurements when evaluating COVID-19 pathogenesis [19,20,21].

The aim of this study was to investigate serum IgE, IgG, and the counts of eosinophils and basophils, as well as the levels of interleukin-10 (IL-10) and interleukin-33 (IL-33), in COVID-19 patients using fresh and frozen serum samples collected during the pandemic. Additionally, we quantified mast cells, basophils, and plasma cells in lung tissue specimens from patients who died of COVID-19 during the same period.

## 2. Materials and Methods

### 2.1. Study Design

This retrospective study included 21 patients who experienced severe complications of COVID-19. Cases were managed at three hospitals in Plovdiv, Bulgaria: St George University Hospital, University Hospital Kaspela, and St Panteleimon Municipal Hospital. Autopsy material was collected between 28 February 2020 and May 2022.

A total of 21 patients (12 males, 56.4%; 9 females, 43.6%) aged 41–78 years (mean 59.4) were included. Most patients had severe cases of COVID-19 (*n* = 13, 61.9%), while four were mild (19.2%) and four moderate (19.2%). All patients had comorbidities, the most common being arterial hypertension (AH) (*n* = 10, 38.1%). Chronic kidney disease (CKD) and autoimmune conditions were also common. Patients were stratified into two groups according to the phase of illness: first group (acute phase, ≤17 days from onset; *n* = 8 patients) and second group (post-acute phase, >20 days from onset; *n* = 13 patients). In total, 21 patients were included.

The laboratory results and clinical characteristics of the COVID-19 patients are available in Supplementary Table S1.

### 2.2. Vaccination Status

Among the five vaccinated patients (all with autoimmune diseases), two developed severe post-COVID lobar pneumonia, while three experienced exacerbations of their underlying autoimmune conditions. Most patients were unvaccinated (recorded as “no”). Only five patients had received Pfizer-BNT162b2 vaccination (Pfizer Inc., New York, NY, USA; two to three doses), and all of them had concomitant autoimmune diseases. Vaccinated patients developed either lobar pneumonia or exacerbations of pre-existing immune disorders, including glomerulonephritis, granulomatous polyangiitis, and systemic lupus erythematosus with lupus nephritis.

### 2.3. Participants and Sample Collection

Venous blood samples were collected from COVID-19 patients. After venipuncture, the serum was separated within 4 h and stored at −80 °C until further use. Of the 21 serum samples, eight were stored at −80 °C until analysis, while the remaining 13 were analyzed fresh.

Blood samples were centrifuged at 1000× *g* for 10 min using a Shimadzu UV160A centrifuge (S. No: 28006648, Kyoto, Japan), after which sera were collected and stored at −80 °C. Before analysis, the thawed homogenates were centrifuged at 3000 rpm for 5 min at +4 °C (Sigma 3K30, S. №: 76262, Sigma Laborzentrifugen GmbH, Osterode am Harz, Germany), and the supernatants were harvested for biochemical assays.

Peripheral blood mononuclear cells (PBMCs) were obtained from whole blood via density gradient centrifugation using Histopaque^®^-1077 (Merck KGaA, Darmstadt, Germany). The isolated cells were resuspended in freezing medium, stored in cryotubes at −80 °C Nalgene^®^ Mr. Frosty (Thermo Fisher Scientific Inc, Waltham, MA, USA), and subsequently transferred to liquid nitrogen for long-term storage.

### 2.4. Laboratory Analyses

Total IgE was quantified using chemiluminescence immunoassay (CLIA) on a Maglumi X3 Vi analyzer (Snibe Diagnostics, Shenzhen, China). Other immunoglobulins were measured via immunoturbidimetry on a BA200 biochemical analyzer (BioSystems, Carrer de la Costa Brava 30, 08030 Barcelona, Spain). The standard reference value for the total serum IgE was <100 IU/mL, while the reference values for the remaining immunoglobulins are provided in Table 1. Complete blood counts (CBC) and differential blood counts (DBC) were performed using standard hematological methods. For patient №15, their CBC and DBC values were obtained in an external laboratory, where the reference range for eosinophils was 30–350 cells/L.

### 2.5. Enzyme-Linked Immunosorbent Assay (ELISA)

Humoral biomarkers (IL-10, IL-33) were assessed via ELISA (SunRed Biological Technology Co., Shanghai, China), and absorption was measured at 450 nm on a BioTek^®^ 800™TS bioreader (BioTek Instruments, Inc., Winooski, VT, USA).

### 2.6. Autopsy Procedure

Samples were obtained from both lungs, including the central and peripheral regions of the lobes, as previously described in an earlier publication on the autopsied patients (Genova et al., 2023) [22]. Four paraffin blocks were collected from each lung for routine examination with hematoxylin and eosin (H&E) staining. Autopsy materials were fixed in 10% neutral-buffered formalin, processed using standard protocols, and stained with H&E (Hematoxylin, No. HT40; Eosin, No. HT110; Sigma Chemical, St. Louis, MO, USA). Histochemical analyses were performed on samples derived from the same blocks. Examination was stratified according to the stage of pneumonia: acute phase (diffuse alveolar damage, DAD), ARDS, organizational phase, and fibrotic phase. Additional staining with Toluidine Blue was performed. Purple-stained basophilic cells were identified at 400× magnification. Semi-quantitative assessments were independently performed by two pathologists. Images were acquired using a Nikon Eclipse 80i microscope equipped with a digital camera and NIS-Elements Advanced Research Software (version 4.13; Nikon Instruments, Tokyo, Japan).

### 2.7. Histopathological and Immunohistochemical Testing

Paraffin blocks of lung tissue from 30 autopsies were processed for hematoxylin and eosin (H&E) and Toluidine Blue staining. Immunohistochemistry (IHC) was performed on formalin-fixed, paraffin-embedded 5 μm sections. Sections were incubated at 90 °C for at least 20 min, followed by deparaffinization. The immunohistochemical analysis of both lungs was performed using anti-CD117 (c-Kit) (mast cell marker, clone T595, Leica Biosystems, Newcastle upon Tyne, UK, Cat# CD117LCE, (RRID: AB_442049)) and anti-CD138 (plasma cell marker, clone OC2, Leica Biosystems, Cat# PA0088, Bond RTU Primary Antibody, (RRID: AB_10555989)). IHC staining was performed with an Autostainer Leica BOND–Automated IHC and ISH Staining System.

### 2.8. Statistical Analysis

Quantitative data were analyzed using the GraphPad Prism software (GraphPad Software 8.0.1 version, Inc., La Jolla, CA, USA). Results are presented with the mean and standard deviation (mean ± SD). Data was processed and analyzed using Microsoft Excel (Microsoft Corp., Redmond, WA, USA). Descriptive statistics, including mean values and percentages (%), were calculated to summarize the results. When applicable, variability was expressed as standard deviation (SD). Data presentation and calculations were performed using Excel functions to ensure accuracy, transparency, and reproducibility. No inferential statistical tests were applied, as the analysis focused solely on the descriptive characterization of the dataset.

## 3. Results

### 3.1. Clinical Characteristics

A total of 21 patients (12 males, 56.4%; 9 females, 43.6%) aged 41–78 years (mean 59.4) were included. Most patients had severe cases of COVID-19 (*n* = 13, 61.9%), while four were mild (19.2%) and four moderate (19.2%). All patients had comorbidities, the most common being arterial hypertension (AH) (*n* = 10, 38.1%). Chronic kidney disease (CKD) and autoimmune conditions were also common.

Eleven patients had different forms of glomerulopathies. Seven patients had been diagnosed with a glomerulopathy one to twenty years before COVID-19 infection, and six of these were on corticosteroid and immunosuppressive therapy, except for one with diabetic nephropathy. The patients’ immunoglobulin levels were monitored periodically, with their IgE values within the normal range before COVID-19 infection. Patients with chronic allergic and immune diseases (various glomerulopathies) were on maintenance therapy with corticosteroids and immunosuppressants. Despite this supportive therapy, five patients developed elevated IgE levels after COVID-19 infection and experienced the exacerbation of pre-existing chronic diseases, requiring hospitalization and full clinical evaluation.

Glomerulopathy in five patients (patients 12, 14, 18, 19, and 20) was diagnosed two to four months after COVID-19 infection, suggesting a possible causal association.

All patients received anticoagulant therapy. Upon admission, patients with severe COVID-19 presented with elevated D-dimer and fibrinogen levels, which normalized after treatment. Patients with severe disease, low oxygen saturation, and respiratory failure also received oxygen therapy.

### 3.2. Serum IgE and Immunoglobulins

Elevated IgE levels (>100 IU/mL) were detected in 10/21 patients (47.6%); two of them had levels more than >1000 IU/mL.

In the 1st group (*n* = 8), four patients (50%) demonstrated elevated IgE, one of whom had levels exceeding 1000 IU/mL.

In the 2nd group (*n* = 13), six patients (46.2%) had elevated IgE, one of whom had markedly increased levels (1721.7 IU/mL).

Serum IgG concentrations were within the normal range in nearly half of the patients (10/21, 47%), reduced predominantly in severe cases (9/21, 42.3%), and elevated in only two cases. The patients’ eosinophil and basophil counts were generally reduced in severe cases, except for patients with lobar pneumonia, who exhibited increased values. Measurements of immunoglobulins, interleukins, eosinophils, and basophils were obtained longitudinally between day 9 and day 90 of illness, enabling the stratification of patients into acute and post-acute groups.

### 3.3. Cytokines

The patients’ IL-33 levels were consistently reduced, both in severe forms of COVID-19 and in patients with long COVID. In contrast, IL-10, an anti-inflammatory cytokine, was significantly elevated in most severe cases (9/10, 42.9%); for example, patient No. 2 exhibited a level of 223.2 pg/mL. These findings suggest that reduced IL-33 may be associated with disease severity and persistence, while elevated IL-10 likely reflects a compensatory anti-inflammatory response.

### 3.4. DBCs

The measured values for eosinophils (Eo) and basophils (Baso) showed that most severe cases had reduced levels of serum eosinophils (<0.05), which are typical of viral infections, including COVID-19 [21,22].

Exceptions were patients 15 and 16, who had elevated levels of eosinophils and basophils, likely related to lobar pneumonia (Supplementary Table S1; Table 1).

Table 1 summarizes the patients’ immunological and hematological parameters stratified by vaccination status. In the unvaccinated group, IgE values ranged widely (from low to >1000 IU/mL) and were frequently accompanied by reduced IgG; IL-10 and IL-33 were elevated in severe cases. In vaccinated patients, IgG levels were closer to the reference range with only moderate increases in IgE, and IL-10/IL-33 appeared more stable. Overall, unvaccinated individuals displayed a more unstable immune profile and higher inflammatory markers, whereas vaccinated patients showed a more balanced immune response.

**Figure 1:**
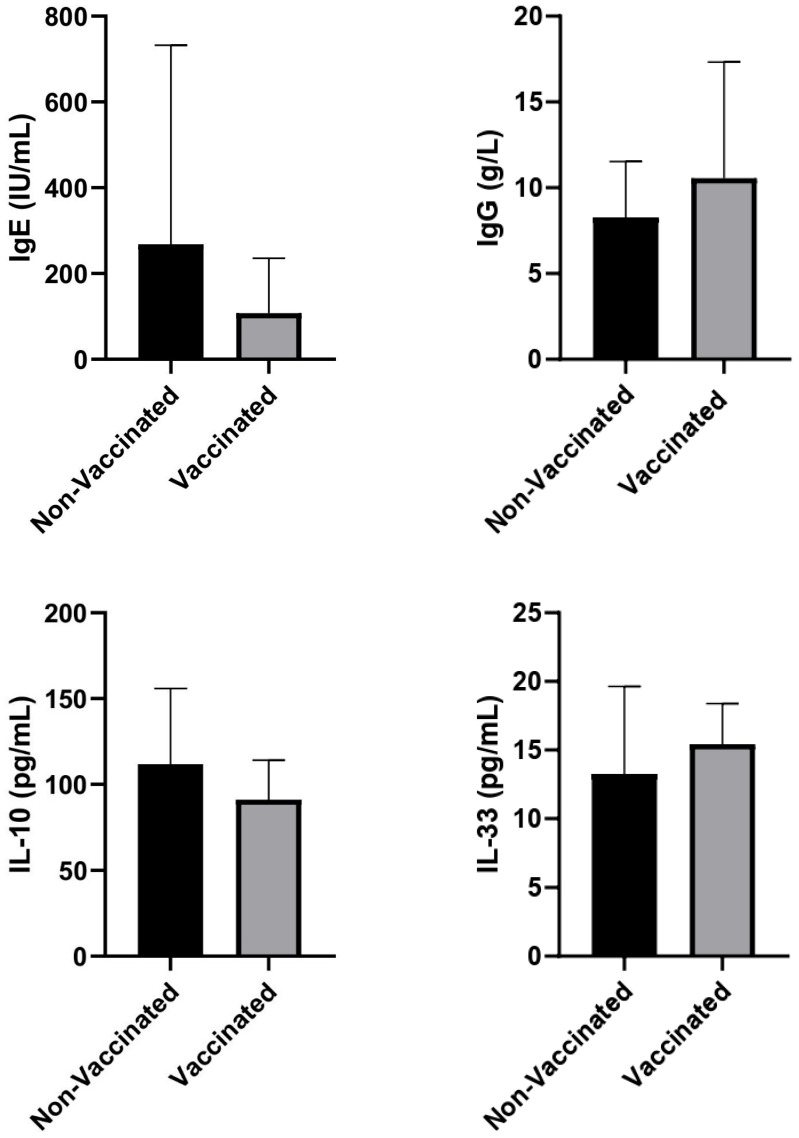
Comparison of IgE, IgG, IL-10, and IL-33 serum levels between vaccinated and non-vaccinated patients.

When comparing unvaccinated and vaccinated individuals, differences were found in some immunological indicators. IgE levels were higher in the unvaccinated group, with a wider range of variation; meanwhile, IgE levels were lower and more concentrated around the mean in the vaccinated group (Figure 1). Conversely, IgG tended to be higher among the vaccinated group compared to the unvaccinated group. The concentrations of interleukin-10 (IL-10) showed a slight decrease in the vaccinated group compared to the unvaccinated group, although they had similar standard deviation limits. Regarding interleukin-33 (IL-33), the values were similar in both groups, without any clear differences.

The results showed higher levels of IgE in the unvaccinated group and higher levels of IgG in the vaccinated group, while IL-10 and IL-33 remained relatively similar between the groups (Figure 1).

**Figure 2:**
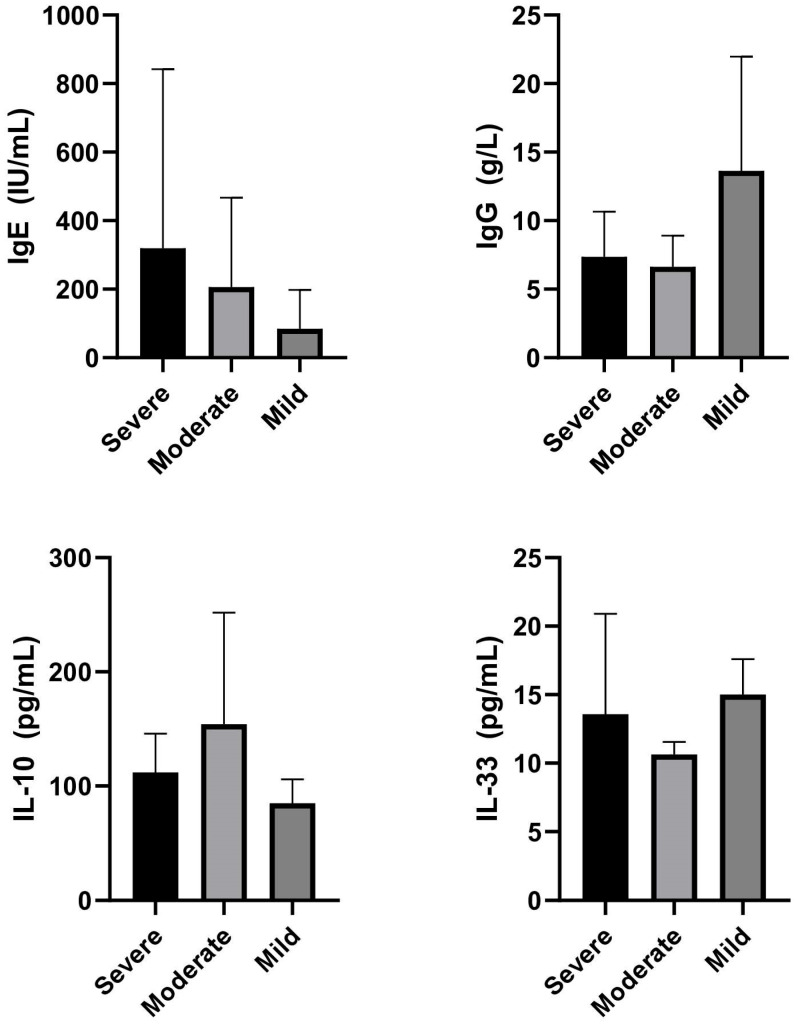
Comparison of IgE, IgG, IL-10, and IL-33 levels in patients with severe, moderate, and mild COVID-19.

Patients with severe COVID-19 exhibited the highest mean IgE levels (319.2 ± 522.7 IU/mL), accompanied by elevated IL-10 concentrations (111.8 ± 34.0 pg/mL) (Figure 2). The mean IgG values in this group were 7.36 ± 3.30 g/L, while IL-33 levels reached 13.6 ± 7.3 pg/mL (Figure 2).

In the moderate group, IgE levels were also elevated (206.4 ± 260.7 IU/mL), with IL-10 showing the highest mean concentration among all groups (154.1 ± 97.7 pg/mL). The mean IgG and IL-33 values were 6.64 ± 2.28 g/L and 10.6 ± 0.9 pg/mL, respectively.

Patients with the mild form of COVID-19 demonstrated significantly lower IgE values (84.5 ± 113.2 IU/mL), while IgG reached comparatively higher levels (13.6 ± 8.3 g/L). The mean IL-10 and IL-33 concentrations in this group were 84.8 ± 21.2 pg/mL and 15.0 ± 2.6 pg/mL, respectively (Figure 2).

Overall, these results suggest that severe COVID-19 is associated with elevated IgE and IL-10 levels, whereas mild disease is characterized by lower IgE and relatively higher IgG concentrations. The moderate form shows an intermediate profile but is distinguished by a pronounced increase in IL-10 (Figure 2).

### 3.5. Histopathology and Immunohistochemistry

**Figure 3:**
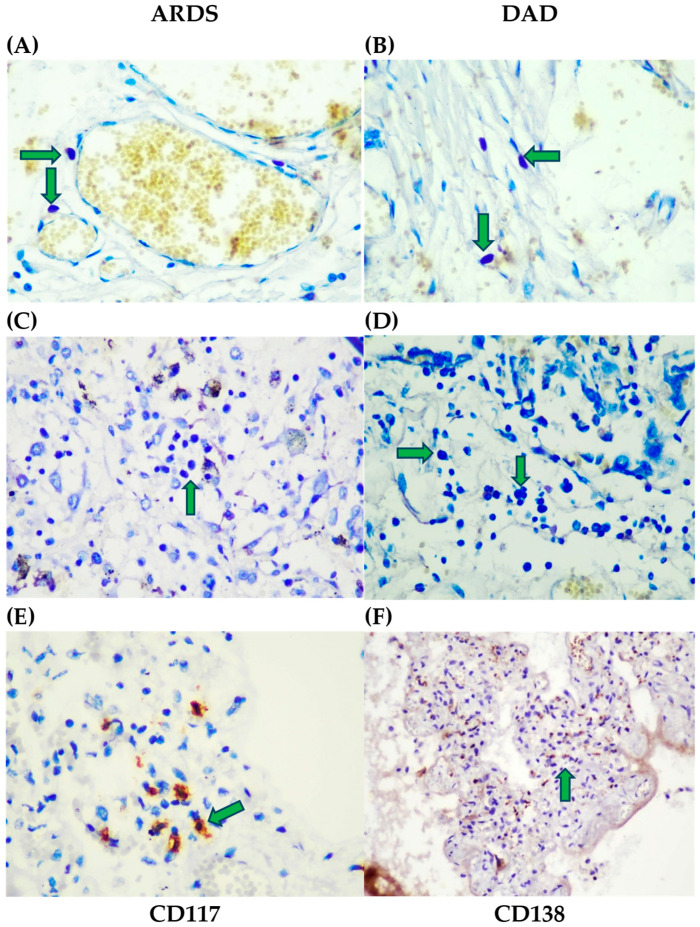
Light microscopy of lung tissue in ARDS and DAD. (**A**,**B**) Basophil cells (arrows) are observed perivascularly, subendothelially and in the stroma in ARDS and DAD pneumonia (Toluidine blue, ×400). (**C**,**D**) Plasma cells (arrows) observed in ARDS and DAD pneumonia (Toluidine blue, ×200). (**E**) Lung parenchyma in COVID-19 pneumonia showing groups of mast cells in the alveolar walls, immunohistochemistry (IHC), CD117 marker, cytoplasmic expression (arrows), ×400. (**F**) Plasma cells infiltrating the alveolar walls: CD138 marker, cytoplasmic expression (arrows), IHC quantification of plasma cells, ×200.

In acute DAD/ARDS, the immunohistochemical analysis revealed the presence of mast cells (CD117^+^) and plasma cells (CD138^+^), ranging from 5 to 20 per high-power field (HPF), as well as basophils, which were consistently detected at 3–5 cells per HPF. Basophils were localized both within the interstitial tissue and in close proximity to the capillary basal membranes or immediately beneath the endothelium. In contrast, mast cells, plasma cells, and basophils were absent during the organizational and fibrotic stages (Figure 3). Negative control lung tissue from a 43-year-old patient who died in a car accident likewise showed no mast cells or basophils.

Toluidine blue staining reveals characteristic cellular infiltrates localized in various structural compartments of the lung (Figure 3). In ARDS and DAD pneumonia preparations, basophilic cells (arrows) are observed to be located perivascularly, subendothelially and in the stroma. These cells are identified by the intense metachromasia of their granules and by their characteristic position relative to the vascular structures (Figure 3A,B, arrows). The presence of basophils in the perivascular spaces suggests an active inflammatory process and participation in the mediator cell response.

In the same groups of patients, plasma cells (arrows) are visualized, localized diffusely in the interstitium and walls of the alveoli. They are characterized by an eccentrically located nucleus and basophilic cytoplasm (Figure 3C,D, arrows). This indicates active local antibody production and an enhanced humoral immune response in ARDS and DAD pneumonia.

In lung tissue preparations from COVID-19 pneumonia, clusters of mast cells (6–8 cells each) localized in the alveolar walls are detected. Immunohistochemistry with CD117 demonstrates a cytoplasmic expression that clearly delineates mast cells (Figure 3E, arrows). The presence of mast cell clusters reflects their role in the pathogenesis of SARS-CoV-2-induced alveolar damage.

In the alveolar walls in the same cases, the significant infiltration of plasma cells is recorded. Immunohistochemistry with CD138 labels them cytoplasmically, allowing the reliable quantification of the plasma cell population (Figure 3F). This is evidence of a pronounced local immune response with a high production of immunoglobulins in the lung tissue.

In summary, observations from light microscopy macrophotographs show the combined presence of basophils, mast cells, and plasma cells in the lung tissue of ARDS, DAD, and COVID-19 pneumonia. This cellular infiltrate is indicative of a complex inflammatory and immunological response involving both innate and adaptive immunity, with potential relevance to alveolar–capillary barrier damage.

## 4. Discussion

The first study reporting that SARS-CoV-2 induces an IgE response—and that serum IgE levels positively correlate with disease severity, suggesting a link between elevated antibody levels and mast cell activation—was conducted by Jānis Plūme et al. (2022) [23]. This study also demonstrated that SARS-CoV-2 triggers IgE responses against the N, S, and M proteins in the sera of COVID-19 patients, with IgE acting as a classical mast cell activator [7]. The increase in IgE production is often associated with natural exposure to the corresponding allergen.

In contrast, IgG4 appears to play a regulatory role in this context [24]. Via direct competition, IgG4 can prevent IgE from binding allergens in the serum [25,26], and more importantly, it directly suppresses IgE-mediated mast cell activation, thereby mitigating downstream allergic and inflammatory responses [8,27,28].

Rubio-Casillas et al. (2025) expanded on this hypothesis, proposing that SARS-CoV-2 presents “allergen-like” epitopes that induce IgE synthesis to evade the immune system and establish chronic infection [7,29]. In response, the immune system produces IgG4 antibodies, which we previously demonstrated in the lungs of patients who died from COVID-19 [30]. These antibodies likely increased to neutralize the deleterious effects of IgE [29,31].

Elevated IgG4 levels are more plausibly interpreted as a downstream effect of severe disease rather than a primary determinant of mortality [15]. The fatal outcome in these patients may, at least in part, reflect IgE-mediated allergic responses targeting the N and Spike proteins of SARS-CoV-2. Within this framework, IgG4 synthesis could represent an adaptive counter-regulatory mechanism aimed at dampening hyperinflammation and mitigating cytokine storm [29].

Taken together, the data supports the hypothesis that SARS-CoV-2 has evolved epitopes within the N and Spike proteins that function as allergens, promoting the production of IgE; this undermines antiviral defenses, while simultaneously triggering the induction of IgG4 as a compensatory response [7,13,29].

In our study, the highest IgE concentrations were detected in patients with pronounced immune activation; for example, the concentration of IgE in patient No. 12 reached 1721.7 IU/mL, suggesting the involvement of allergic or autoimmune mechanisms. By contrast, the IgE levels in mild cases remained within or only slightly above the reference ranges. The serum IgG values were largely normal; however, in cases of severe disease, the IgG concentrations were frequently reduced (e.g., patient No. 3, 5.766 g/L), likely reflecting advanced disease-associated immunosuppression. Recent reports also show that IgG4 competes with specific IgE for allergen binding as a blocking antibody, preventing the degranulation of mast cells and basophils [13,14,29]. Another possibility is that in severe COVID-19 pneumonia, SARS-CoV-2 induces IgG4 synthesis in an IgE-independent manner by inhibiting the binding of IgG3 to its Fc receptor, thereby impairing viral phagocytosis [32,33].

High concentrations of free IgE can activate mast cells similarly to antigen-bound IgE, amplifying immune responses even without antigen stimulation [34]. Regarding COVID-19, a study by Körner, R.W. et al. (2023) examined 70 pediatric patients, finding sensitization to aeroallergens in 93% of cases [18].

Elevated IgE levels (meaning 174.2 kU/L) were observed to be independent of disease severity. Consistent with recent observations, IgE elevation emerges as a consistent feature of post-COVID syndrome in both allergic and non-allergic individuals [14,35]. These findings suggest that atopic patients with pre-existing IgE elevation are particularly prone to aggravated post-viral immune dysregulation. Moreover, our results indicate that SARS-CoV-2 infection exacerbates underlying immune and autoimmune disorders, irrespective of the initial disease severity [36,37].

Our study also found that existing immune and autoimmune diseases worsened post-COVID infection, regardless of disease severity. In another study by Guclu et al. (2022), involving 202 patients, the total serum IgE concentrations were significantly higher (mean 172.90 IU/mL), while the serum eosinophil levels were significantly lower (mean 0.015/mL) [1]. The two patients with lobar pneumonia and high IgE levels in our cohort had mild COVID-19 symptoms initially, but pneumonia developed suddenly one month after recovery. Both patients had been diagnosed with allergic diseases. Five patients had elevated IgE levels alongside immune or autoimmune disorders, including immune glomerulonephritis, Hashimoto’s thyroiditis in remission, and bronchial asthma.

All were admitted with exacerbations of their underlying conditions.

Patients with allergic conditions were in remission under maintenance therapy, and their laboratory tests, including IgE, were within normal ranges prior to SARS-CoV-2 infection. Nearly half of the patients had serum IgG concentrations within reference limits. The reduced IgG levels observed in severe COVID-19 are most likely attributable to disease-associated immunosuppression, whereas the elevated levels observed in cases of lobar pneumonia appear to be linked to concomitant allergic conditions.

The patients’ serum IgG levels were within normal limits, except in the two patients with lobar pneumonia. Elevated IgE levels were observed in almost half of the patients. These values correlated with the decreased eosinophil and basophil levels observed in peripheral blood, except in those with post-COVID lobar pneumonia, where eosinophils were elevated and basophils normal. Normal levels of IgG were observed in patients with COVID-19 pneumonia and elevated levels were observed in patients with lobar pneumonia [38].

Regarding the efficacy of vaccination, recent publications show that natural COVID-19 induces IgE, but vaccination raises its levels further [9,14]. IgE demonstrates moderate to high avidity, particularly after booster vaccination. The levels of IgG4 antibodies also increase, mainly post-booster, and moderately correlate with IgE [14]. COVID-19 appears to induce IgE with intermediate avidity, whereas vaccines primarily drive high-avidity IgE [8].

Other studies report associations between elevated proinflammatory cytokines and mRNA vaccines [39], but most patients were unvaccinated in our cohort. Two patients with pre-existing allergic conditions who had received three booster doses developed severe lobar pneumonia one month after mild COVID-19. Three other vaccinated patients experienced exacerbations of glomerulonephritis, granulomatous polyangiitis, and systemic lupus erythematosus with lupus nephritis.

Cytokine profiling revealed distinct signatures: IL-10 was markedly elevated in severe cases, particularly in patients with pulmonary inflammation and in complicated cases beyond day 25, consistent with an anti-inflammatory counter-regulatory response. In contrast, IL-33 was consistently reduced in both severe COVID-19 and post-acute sequelae (Long COVID). The nearly universal elevation of IL-10 in severe disease suggests the host’s attempt to restrain hyperinflammation. These molecular observations are corroborated by large-scale electronic health record analyses, which demonstrate that asthma confers a higher risk of hospitalization and mortality in COVID-19 compared with other allergic conditions such as atopic dermatitis [9]. Likewise, national cohort studies from South Korea, Japan, and the UK Biobank indicate that the risk of new-onset allergic diseases rises significantly within the first 30 days after COVID-19, particularly in patients with asthma and allergic rhinitis [10]. Although attenuating over time, this risk persists for at least six months and correlates with the initial severity of the disease [10].

Basophils and mast cells are key inducers of Th2 responses and play critical roles in both allergic and antiparasitic immunity, as well as in antiviral defense [10,11].

Basophils can also function as antigen-presenting cells, binding antigens on their surface and promoting humoral immunity via Th2 cell differentiation [12,40].

Previous studies have reported reduced circulating basophil counts during the acute and severe phases of COVID-19 [11]. In line with these findings, we observed decreased serum eosinophil and basophil levels in most patients. In striking contrast, however, lung tissue from deceased patients demonstrated the marked accumulation of mast cells, basophils, and plasma cells, suggesting that peripheral depletion reflects recruitment into inflamed tissues [41].

During the early organization and fibrosis phases, basophils and plasma cells were absent.

Taken together, these findings indicate that COVID-19 is associated with a shift toward IgE and IgG4 production, accompanied by the peripheral depletion but tissue accumulation of basophils and mast cells. This immunological profile suggests the redistribution of effector cells to inflamed lung tissue and a dysregulated antibody response that may contribute to both acute pathology and post-acute sequelae.

Our study has several limitations. The relatively small sample size limits the generalizability of the findings. Access to material from severe COVID-19 cases was also restricted, as it was only obtained from patients treated in intensive care settings, which may not reflect the full spectrum of the diseases.

Another limitation is the small number of vaccinated and healthy participants. This was influenced by the prevailing hesitancy toward vaccination at the time of recruitment. In addition, the unequal gender distribution further restricted subgroup analyses and hampered our ability to examine potential sex-related differences.

A further limitation is that the IgE antibodies measured in serum were total IgE; however, the clinical data obtained provide a rationale for subsequent studies of SARS-CoV-2–specific IgE antibodies.

Future studies with larger and more balanced cohorts are needed to confirm and extend these findings.

## 5. Conclusions

Our findings indicate that SARS-CoV-2 infection may elicit an IgE-mediated, allergy-like immune response that contributes to both the severity of acute disease and long-term post-COVID-19 immune dysregulation. Elevated IgE levels were common and associated with mast cell activation, persistent inflammation, and exacerbations in patients with pre-existing allergic or autoimmune conditions. In contrast, high IgG4 levels may exert a counter-regulatory effect, mitigating IgE-driven responses. The observed cytokine profiles (increased IL-10, reduced IL-33) further support a dysregulated immune balance. Taken together, our findings suggest that monitoring IgE and mast cell activity may provide valuable insights into the immunopathogenesis of COVID-19, while IgG4 could represent a potential counter-regulatory factor.

However, given the limited sample size of our study, these conclusions should be considered preliminary.

Future large-scale, well-controlled mechanistic studies are essential to validate these observations, clarify the underlying pathways, and determine their therapeutic implications in both acute and post-acute COVID-19 syndromes.

## Figures and Tables

**Table 1:** Hematological and immunological values in non-vaccinated individuals and vaccinated individuals.

No.	IgE (IU/mL)	IgG (g/L)	IL-10 (pg/mL)	IL-33 (pg/mL)	Eo (10^9^/L)	Baso (10^9^/L)	Ly (10^9^/L)	PMN (10^9^/L)	Ly/PMN Ratio %
Non-Vaccinated individuals									
1	114	5.934	110.3	21.34	0.01	0.02	0.52	5.52	9.8
2	123	13.8	203.4	3.72	0.01	0.01	0.62	5.23	11.3
3	1002	5.766	114.4	20.95	0.01	0.03	0.63	10.14	5.9
4	107	5.841	109	22.41	0.00	0.04	0.74	6.17	10.9
5	16.5	9.354	113	3.41	0.01	0.02	2.31	3.26	36.8
6	41.9	6.875	121.5	5.978	0.07	0.20	1.11	4.92	17.0
7	22	8.248	223.2	11.28	0.02	0.03	3.30	4.25	21.7
8	45	11.38	139.6	24.55	0.05	0.02	1.24	3.72	18.2
9	5.97	6.54	75	15	0.03	0.01	0.6	5	29.8
12	1721.7	2.33	88	12	0.04	0.03	1.4	7.2	4.5
13	271.1	9.82	90	11	0.03	0.02	1.5	2.6	34.7
14	378	8.537	92	10.5	0.04	0.03	2.1	6.7	22.8
17	390.7	5.025	85	10	0.03	0.02	1.7	3	19.2
19	55.67	13.85	73	14	0.03	0.01	2.2	4.6	31.2
20	1.19	7.072	74	13	0.01	0.01	1.3	5.3	18.1
21	1.426	11.95	76	13	0.02	0.01	1.7	4.8	27.0
Vaccinated individuals									
10	5.443	3.843	78	14	0.02	0.01	0.45	10.08	4.5
11	4.775	4.887	82	13	0.03	0.02	2.14	11.52	18.6
15	276	16.81	131.7	19.43	0.423	0.5	1.50	11.52	13.0
16	215	18.54	86.95	17.69	1.2	0.6	0.74	7.20	10.3
18	36.15	8.67	77	13	0.02	0.01	2.40	10.08	23.8

Legend: IgE (IU/mL)—serum immunoglobulin E levels; IgG (g/L)—serum immunoglobulin G levels; IL-10 (pg/mL)—interleukin-10; IL-33 (pg/mL)—interleukin-33; Eo (10^9^/L)—eosinophils (count per liter); Baso (10^9^/L)—basophils (count per liter); Ly/PMN ratio—lymphocyte-to-neutrophil ratio.

## Data Availability

The original contributions presented in this study are included in the article/Supplementary Material. Further inquiries can be directed to the corresponding author.

## Supplementary Materials

The following supporting information can be downloaded at: https://www.mdpi.com/article/10.3390/life15101538/s1, Table S1: Clinical characteristics.
