# Supplementary material for: High Free IgE and Mast Cell Activation in Long COVID: Mechanisms of Persistent Immune Dysregulation
Source: Life (Basel). 2025 Oct 1;15(10):1538. doi: 10.3390/life15101538 (PMC12565182; doi:10.3390/life15101538)
Supplement: Supplementary file 1 [file life-15-01538-s001.zip › life-3873492-supplementary.pdf]

No.	Sex	Age	Form of illness	Day of illness	Disease	Pharmacological treatment prior infection	Pharmacological treatment during infection	Lung function SpO2 (T)	IgE <100 IU/ml	IgG 7.0–16.0 g/l	IL-10 3–7 pg/ml	IL-33 50–62 pg/mL	Eo 0.04 - 0.4 (10^9/l)	Baso 0.5% -1% (10^9/l)	Vaccines	Comorbidities
1	m	71	Severe	7	Deceased COVID-19 pneumonia	Antihypertensive therapy	AB, Corticosteroid	60%	114	5.934	110.3	21.34	0.01	0.02	no	AH, IHD
2	m	68	Severe	9	COVID-19 pneumonia	Antihypertensive therapy	AB, Corticosteroid	87-93%	123	13.8	203.4	3.72	0.01	0.01	no	AH
3	f	77	Severe	11	COVID-19 pneumonia	no	AB, Corticosteroid	89-93%	1002	5.766	114.4	20.95	0.01	0.03	no	Kidney cancer, stage IV
4	m	49	Severe	14	COVID-19 pneumonia	no	AB, Corticosteroid	83-89%	107	5.841	109	22.41	0.00	0.04	no	Not reported
5	m	57	Severe	16	COVID-19 pneumonia	Antihypertensive drug	AB, Corticosteroid	89-94%	16.5	9.354	113	3.41	0.01	0.02	no	AH
6	f	78	Severe	16	COVID-19 pneumonia	Insulin	AB	87-92%	41.9	6.875	121.5	5.978	0.07	0.20	no	Diabetes
7	m	59	Moderate	17	COVID-19 pneumonia	Antihypertensive therapy	AB, Corticosteroid	89-93%	22	8.248	223.2	11.28	0.02	0.03	no	AH
8	f	74	Severe	17	COVID-19 pneumonia	Insulin	AB	88-91%	45	11.38	139.6	24.55	0.05	0.02	no	Diabetes
9	f	47	Mild	20	Rhino-pharyngitis	Corticosteroid; immunosuppressive therapy, Rituximab	AB, Corticosteroid; immunosuppressive therapy	90-97%	5.973	6.54	75	15	0.03	0.01	no	GPA, AH, chronic lung disease
10	m	64	Severe	20	COVID-19 pneumonia, secondary infection	Corticosteroid; immunosuppressive therapy, Rituximab	AB, Corticosteroid; immunosuppressive therapy	80-86%	5.443	3.843	78	14	0.02	0.01	3	GPA, AH, chronic lung disease
11	f	54	Severe	22	COVID-19 pneumonia	Corticosteroid; immunosuppressive therapy, Rituximab	AB, Corticosteroid; immunosuppressive therapy	88-92%	4.775	4.887	82	13	0.03	0.02	3	SLE, lupus nephritis, AH, osteoporosis
12	m	50	Severe	25	COVID-19 pneumonia	no	AB, Corticosteroid;	88-92%	1721.7	2.33	88	12	0.04	0.03	no	Membranous GN
13	m	57	Severe	28	COVID-19 pneumonia	Corticosteroid; immunosuppressive therapy	AB, Corticosteroid;	80-88%	271.1	9.82	90	11	0.03	0.02	no	IgA nephropathy, HTN, DM, gout

14	m	59	Severe	30	Bilateral pneumonia	no	AB, Corticosteroid;	86-93%	378	8.537	92	10.5	0.04	0.03	no	CKD, AH
15	f	56	Mild	30	Crupous pneumonia	no	AB	93-95%	276	16.81	131.7	19.43	0.423	0.5	3	Hashimoto thyroiditis (remission)
16	f	58	Mild	32	Crupous pneumonia	Inhaled corticosteroids	AB, Inhaled corticosteroids	93-95%	215	30.54	86.95	17.69	1.2	0.6	3	Bronchial asthma
17	m	64	Moderate	40	COVID-19 pneumonia	Insulin	AB, Corticosteroid;	82-98%	390.7	5.025	85	10	0.03	0.02	no	AH, diabetes, diabetic nephropathy
18	m	41	Mild	42	Rhino-pharyngitis	no	AB, Corticosteroid;	94-98%	36.15	8.67	77	13	0.02	0.01	2	Membranous GN, AH, hypothyroidism
19	f	58	Mild	60	Rhino-pharyngitis	no	AB, Corticosteroid;	94-98%	55.67	13.85	73	14	0.03	0.01	no	CKD, AH
20	m	59	Mild	90	Rhino-pharyngitis	no	AB, Corticosteroid;	99.0%	1.19	7.072	74	13	0.01	0.01	no	Membranous GN, AH
21	f	47	Mild	90	Rhino-pharyngitis	Corticosteroid, immunosuppressive therapy	AB, Corticosteroid; immunosuppressive therapy	97-99%	1.426	11.95	76	13	0.02	0.01	no	Microscopic polyangiitis, hypothyroidism

Table S1. Clinical characteristics

Legend: AH (Hypertension); IHD (Ischemic Heart Disease); DM (Diabetes Mellitus); CKD (Chronic Kidney Disease); GN (Glomerulonephritis); SLE (Systemic Lupus Erythematosus).
